# Assessing viability and infectivity of foodborne and waterborne stages (cysts/oocysts) of *Giardia duodenalis, Cryptosporidium* spp., and *Toxoplasma gondii*: a review of methods

**DOI:** 10.1051/parasite/2018009

**Published:** 2018-03-19

**Authors:** Angélique Rousseau, Stéphanie La Carbona, Aurélien Dumètre, Lucy J. Robertson, Gilles Gargala, Sandie Escotte-Binet, Loïc Favennec, Isabelle Villena, Cédric Gérard, Dominique Aubert

**Affiliations:** 1 EA 3800, Protozooses transmises par l’alimentation, Laboratoire de Parasitologie Mycologie, Université de Reims Champagne Ardenne, Faculté de Médecine, SFR Cap Santé Fed 4231, 51 Rue Cognacq Jay, 51096 Reims France; 2 ACTALIA Food Safety Department, 310 Rue Popielujko, 50000 Saint-Lô France; 3 Aix Marseille Univ, IRD (Dakar, Marseille, Papeete), AP-HM, IHU-Méditerranée Infection, UMR Vecteurs − Infections Tropicales et Méditerranéennes (VITROME), Marseille France; 4 Department of Food Safety and Infection Biology, Faculty of Veterinary Medicine, Norwegian University of Life Sciences, PO Box 8146 Dep., 0033, Oslo Norway; 5 EA 3800, Protozooses transmises par l’alimentation, Laboratoire de Parasitologie Mycologie, Université de Rouen, 76183 Rouen Cedex France; 6 Food Safety Microbiology, Nestlé Research Center, PO Box 44, CH-1000 Lausanne 26 Switzerland

**Keywords:** Viability, Infectivity, *Cryptosporidium*, *Giardia*, *Toxoplasma*, Risk assessment, Food, Methods

## Abstract

*Giardia duodenalis*, *Cryptosporidium* spp. and *Toxoplasma gondii* are protozoan parasites that have been highlighted as emerging foodborne pathogens by the Food and Agriculture Organization of the United Nations and the World Health Organization. According to the European Food Safety Authority, 4786 foodborne and waterborne outbreaks were reported in Europe in 2016, of which 0.4% were attributed to parasites including *Cryptosporidium*, *Giardia* and *Trichinella*. Until 2016, no standardized methods were available to detect *Giardia, Cryptosporidium* and *Toxoplasma* (oo)cysts in food. Therefore, no regulation exists regarding these biohazards. Nevertheless, considering their low infective dose, ingestion of foodstuffs contaminated by low quantities of these three parasites can lead to human infection. To evaluate the risk of protozoan parasites in food, efforts must be made towards exposure assessment to estimate the contamination along the food chain, from raw products to consumers. This requires determining: (i) the occurrence of infective protozoan (oo)cysts in foods, and (ii) the efficacy of control measures to eliminate this contamination. In order to conduct such assessments, methods for identification of viable (i.e. live) and infective parasites are required. This review describes the methods currently available to evaluate infectivity and viability of *G. duodenalis* cysts*, Cryptosporidium* spp. and *T. gondii* oocysts, and their potential for application in exposure assessment to determine the presence of the infective protozoa and/or to characterize the efficacy of control measures. Advantages and limits of each method are highlighted and an analytical strategy is proposed to assess exposure to these protozoa.

## Abstract (fr)

*Giardia duodenali*s, *Cryptosporidium* spp. et *Toxoplasma gondii* sont des parasites protozoaires qui ont été soulignés comme agents pathogènes émergents dans les aliments par l’Organisation des Nations Unies pour l’alimentation et l’agriculture et l’Organisation Mondiale de la Santé. Selon l’Autorité Européenne de Sécurité des Aliments, 4786 épidémies d’origine alimentaire et hydrique ont été enregistrées en Europe en 2016, dont 0.4% ont été attribuées à des parasites, incluant *Cryptosporidium*, *Giardia* et *Trichinella*. Jusqu’en 2016, aucune méthode standardisée n’était disponible pour détecter les kystes de *Giardia* et les oocystes de *Cryptosporidium* et *Toxoplasma* dans les aliments. Aucune réglementation n’est donc proposée concernant ces dangers. Cependant, compte tenu de leur faible dose infectieuse, l’ingestion d’une quantité d’aliments faiblement contaminés peut entraîner une infection de l’homme. Pour évaluer le risque lié aux protozoaires dans les aliments, des efforts doivent être faits dans l’évaluation de l’exposition pour estimer la contamination le long de la chaîne alimentaire, depuis la matière première jusqu’aux consommateurs. Cette évaluation nécessite de déterminer** **: (i) la prévalence de parasites infectieux dans les aliments, (ii) l’efficacité des mesures de maîtrise pour éliminer cette contamination. Pour mener une telle évaluation, des méthodes capables d’identifier des parasites viables (vivants) et infectieux sont requises. Cette revue décrit les méthodes actuellement disponibles permettant d’évaluer l’infectiosité et la viabilité des kystes de *G. duodenalis* et des oocystes de *Cryptosporidium* spp. et *T. gondii,* et leur potentiel pour être appliquées dans l’évaluation de l’exposition pour déterminer la présence de parasites infectieux et/ou caractériser l’efficacité des mesures de maîtrise. Les avantages et limites de chaque méthode sont présentés et une stratégie d’analyses est proposée pour évaluer l’exposition à ces protozoaires.

## 1 Introduction

*Giardia* *duodenalis* and *Cryptosporidium* spp. are enteric parasites of humans and various other mammals [205,206]. Their cysts and oocysts are usually excreted in high numbers in the feces of infected hosts (e.g., up to 10^9^*Cryptosporidium* oocysts per gram of calf feces) and are immediately infectious upon excretion without requiring a period of development in the environment. Oocysts of the coccidian parasite *Toxoplasma gondii* are shed exclusively in the feces of felids. One infected cat can excrete millions of non-sporulated oocysts shortly after infection [56]. Oocysts become infectious for humans and other warm-blooded animals by sporulation one to five days later, depending on aerobic and temperature conditions [57]. *Giardia duodenalis* cysts, and *Cryptosporidium* spp. and *T. gondii* oocysts can be encountered in different terrestrial and aquatic ecosystems where they can persist for months, possibly years. In addition, these parasites are extremely resistant to many chemical and physical inactivation agents [59]. The cysts and oocysts of these parasites can be transmitted to humans by the fecal-oral route, directly by contact with contaminated hosts (animals or humans depending on the parasites), or indirectly by the waterborne or foodborne routes. Consistent with this, *Cryptosporidium* spp. and *Giardia* spp. accounted for the largest waterborne outbreaks reported between 1998 and 2012 in the European Nordic countries, with more than 50,000 persons infected, although Caliciviruses and *Campylobacter* were the pathogens most frequently involved [95]. Concerning worldwide waterborne outbreaks due to parasitic protozoa, *Cryptosporidium* spp. and *Giardia* spp. are the most commonly reported etiological agents [61]. *T. gondii* oocysts have been responsible for waterborne outbreaks to a lesser extent, being responsible for 2% of parasitic protozoan outbreaks between January 2004 and December 2010 [16,120]. However, due to the usually non-acute nature of infection with *Toxoplasma*, the number of waterborne infections associated with this parasite is probably underestimated. In 2016 in Europe, 4786 food-borne outbreaks, including waterborne outbreaks, were reported, of which 0.4% were due to parasites including *Cryptosporidium*, *Giardia* and *Trichinella* [60]. However, this number may be underestimated considering the large number of outbreaks where the causative agent remains unknown (36%). For the foodborne route, fruits and vegetables, in particular those consumed raw or minimally processed, are probably of greatest relevance [155,175]. Waters used to irrigate fruits and vegetables can be contaminated by *Cryptosporidium* spp., *G. duodenalis,* and *T. gondii* [10,39,147,161,207]. Foods can also be contaminated by oocysts and cysts due to poor hygiene conditions during transformation or preparations, through food handlers, surfaces, or equipment [64,72]. Concerning *T. gondii*, food transmission is more likely through ingestion of cysts in raw or undercooked meat but this is now considered to be a less predominant source of infection in certain areas or populations [31,201].

The low infectious dose for these protozoan parasites means that the associated risk to public health is increased. The ID_50_ (number of oocysts required to infect 50% of exposed people) was estimated to be between 10 and 83 oocysts in healthy adults for *C. hominis* [40], and < 10 to 2000 oocysts for different strains of *C. parvum* [170,204]. Similarly, 10 to 100 cysts of *G. duodenalis* can lead to symptomatic parasitic disease [180]. *Giardia* and *Cryptosporidium* infections cause mild to severe recurrent diarrhea or intestinal disorders, and *Cryptosporidium* is one of the four pathogens involved in most of the cases of diarrhea in children younger than 5 years in low-income countries [130]. For *T. gondii*, the ID_50_ is estimated between 1 and 10 oocysts in rodent models [56]. In immunocompetent individuals, infections are usually asymptomatic, resulting in the formation of latent cysts in tissues and organs throughout the body [182]. Infections can, however, sometimes led to severe ocular, cerebral, or multivisceral complications, especially in congenitally infected infants and in immunocompromized people. Along with the immunity status of the host, the genetic type of *Toxoplasma* is also relevant regarding the development of clinical disease.

Risk assessment in foods includes four components: hazard identification, exposure assessment, hazard characterization, and risk characterization [65]. The oocyst and cyst stages of *Cryptosporidium*, *T. gondii* and *Giardia* are now recognized as hazards in some foodstuffs, and data are available concerning their characterization. Efforts should now focus on determining exposure assessment. This step includes the estimation of the presence of infective protozoa and their amounts in foods along the food chain. For *Cryptosporidium* and *Giardia* (oo)cysts, exposure assessment is partially addressed through the species/genotypes and genetic assemblages, respectively. Indeed, only some species and/or genotypes of *Cryptosporidium* spp. are pathogenic in humans [167,174]. For *G. duodenalis*, only assemblages A and B are responsible for the vast majority of human infections [206]. In addition to these taxonomic data, information on the infectivity of (oo)cysts is also critical to establish the public health significance of protozoan transmission stages in the environment. However, the Standard Method now available for detection of *Cryptosporidium* and *Giardia* in fresh produce [112] is based on immunofluorescence assay microscopy that does not provide this level of information, and neither do other methods that are based on molecular biology detection. These methods have been used to detect these three protozoa in different types of food [4,105,151]. Thus, a rapid and efficient method to distinguish infective (oo)cysts from non-infective (oo)cysts is needed for exposure assessment. Although both *in vitro* and *in vivo* methods have been developed to evaluate the viability and infectivity of the (oo)cysts of these parasites, currently none of these seem to be suitable for routine application in the water and food industries. *In vitro* methods include parasite excystation, fluorogenic dye inclusion/exclusion coupled with DNA amplification or not, ability to invade cells and replicate within, reverse transcriptase-polymerase chain reaction (RT-PCR) of specific gene targets, and fluorescence *in situ* hybridization (FISH). *In vivo* methods consist of animal infectivity assays.

This paper aims to describe the different methods currently available to determine the infectivity and viability of *G.* *duodenalis* cysts, and *Cryptosporidium* spp. and *T.* *gondii* oocysts. An infectious *Giardia* cyst is defined as a viable cyst that is able to release its trophozoites, which can then multiply under appropriate culture conditions. An infectious oocyst of *Cryptosporidium* spp. or *T. gondii* is defined as a viable (oo)cyst that is able to excyst and release its sporozoites, which can then infect host cells and differentiate within into a replicative stage. In comparison, a viable (oo)cyst is defined as being alive but not necessarily infective because its trophozoites/sporozoites would fail to replicate in the host or to cause infection in susceptible cells. The implementation of these methods in the framework of exposure assessment in food is also presented according to two main objectives: i) determining the occurrence of infective parasites in naturally contaminated samples; and ii) determining the efficacy of control measures. Advantages and limitations of each method are described in order to propose an analytical strategy to address component three of risk evaluation in foods, i.e. assessing exposure to these infective parasites.

## 2 Evaluation of infectivity

### 2.2 Bioassay

The bioassay method consists of inoculating (oo)cysts into animals in order to observe an infection. This represents the gold standard to study (oo)cyst infectivity. For each parasite, several models and methods are described. However, as well as being extremely expensive, the use of animals raises major ethical concerns that complicate bioassay applications and should be limited.

#### 2.2.1 *Cryptosporidium spp*.

Animal models, including calves, neonatal mice, pigs and lambs, have shown that *Cryptosporidium* spp. and strains vary in their ability to cause symptomatic infections in various hosts. Models include drug-immunosuppressed and immunodeficient rats [32,179] and mice (SCID, knockout) [21,38,94,156,177,212,230], pigs [101], and gnotobiotic piglets [223,224]. However, the relevance to human infections of infectivity data in animals is unclear. Neonatal mouse models are becoming the reference rodent models for the detection of infectious *C. parvum* oocysts, allowing the determination of the ID_50_ or the lowest infectious dose (LID) required to establish infection. Neonatal BALB/c [11,169] and NMRI suckling mice [49,227] are considered the most appropriate models to evaluate *C. parvum* infectivity, but other models have been used (e.g., CD-1/ICR [73] and ARC/Swiss [102]). By standardizing laboratory methods and data analysis, Korich et al. [128] obtained reproducible results in inter-laboratory trials using CD-1 neonatal mice. The method to assess infectivity involves quantifying the number of oocysts observed in the intestine [73] or in the feces [103]. In terms of sensitivity, for some experiments and models, a single oocyst can be recognized as infectious using this approach [49,228]. However, because bioassays often use different animals or strains of neonatal mice, different strains of *C. parvum,* and/or different methods to detect the infection, such studies have often produced contradictory results [188,194]. Most of the animal models are not susceptible to infection by *C. hominis* or by the other species that sporadically infect humans (particularly immunocompromized individuals) such as *C. felis*, *C. meleagridis, C. canis* and *C. cuniculus*. The only animal models, to date, for *C. hominis* infection are the gnotobiotic pig model [224,225] and the immunosuppressed Mongolian gerbil model [14]. Mouse models allow identification of infective *C. parvum* oocysts in water and shellfish samples, and have been relatively widely used to determine inactivation efficacy of chemical and physical treatments in a simple matrix, but also in different types of food matrices (beverages and solids) (Table 1).

**Table 1:** Methods to determine (oo)cyst infectivity and their application in exposure assessment.

Methods	Parasites	Applications	
		
		Detection in naturally contaminated samples	Determination of the efficacy of control measures^a^
Bioassays − Ability of (oo)cysts to induce infection of animals	Cp	River water [210] Wastewater [109] Mussels, clams, oysters, cockles [67,69,70,71,88,90,143]	**Simple matrix^b^:** H_2_O_2_-based disinfectants [176] Chlorine dioxide [198] Ozone [141,187], ozone + monochloramine/ + chlorine [27,28] Salinity: 10, 20 and 30 ppt of salt at 10 °C or 20 °C (12 w) [68] UV [11,46,148,154,187,195,198] US [172] Pasteurization [99] Storage 15 °C (9 m) [115] Storage 4 °C and 10 °C (8 w) [142] Storage −10 °C to + 35 °C (24 w) [68]
			**Food matrix:** UV − Fresh apple cider [98] PUV − Raspberries [131] HHP, e-beam, microwaves − Oysters [42,43] Heat (steam cooking) − Mussels [91] Pasteurization − Milk [99] Storage 6 °C (4 w) – Apple [150]
	G	Wastewater [82,109,140]	**Simple matrix:**
		Raw and chlorinated drinking water [111]	Ozone [74]
			UV [37,139,140,154,196]
			Gamma irradiation [203]
	T	Water [12,110,219] Oysters, mussels [63]	**Simple matrix:**
			Chlorine [222]
			Ozone [59,222]
			UV [59,220,223]
			HHP [145]
			Gamma-irradiation [55]
			Radiofrequency [221]
			Storage −10 °C to 70 °C (up to 54 m) [53]
			**Food matrix:**
			HHP − Raspberries [146]
			Storage 4 °C (up to 8 w) − Raspberries and blueberries [126]
Cell culture infection −	Cp	Wastewater [85] Water [133,190]	**Simple matrix:**
			Chlorine [15,193], Chlorine dioxide [198], MOS [122]
Ability of (oo)cysts to invade and infect cells			Ozone [122,187]
			UV [83,122,123,186,187,195,198]
			Heat 38 °C to 70 °C [193]
			Storage 15 °C (9 m) [115]
			**Food matrix:**
			Organic acids and hydrogen peroxide − Fruit juices [127]
	Ch	NA	**Simple matrix:**
			UV [118]
	T	NA	**Simple matrix:**
			Chlorine [218]
			Iodophore-based disinfectants, formalin, acidified ethanol [218]
			Ozone [59]
			UV [59,223]

a Relevant for water and food industries;

b Simple matrix: water or buffer.

NA: No available data. Cp: *C. parvum*; Ch: *C. hominis*; G: *Giardia duodenalis*; T: *Toxoplasma gondii*.

H_2_O_2_: hydrogen peroxide; MOS: mixed-oxidant solution; UV: ultraviolet; PUV: pulsed-ultraviolet; HHP: high hydrostatic pressure; US: ultrasound; m: months; w: weeks.

#### 2.2.2 Giardia duodenalis

Few rodent animal models have been described for the evaluation of the infectivity of human isolates of *G. duodenalis*, i.e. genetic assemblages A and B. These assemblages are not naturally able to infect mice and rats [132,232], but inoculation of trophozoites, and not cysts, can still lead to infection of these animals (only neonatal ones, however) [41,137]. Mongolian gerbils appear to be the only model enabling the assessment of cyst infectivity and are also suitable to explore giardiasis symptoms and clinical resistance [18]. In this model, cyst shedding seems discontinuous and infection is usually assessed by determining the presence of trophozoites in the small intestine by microscopy 7 to 10 days after parasite inoculation [2,137]. *G. duodenalis* genetic assemblages A, B and E have all been used to infect gerbils, although assemblage E is less virulent [23]. This technique has been applied to evaluate the efficacy of physical (UV and gamma irradiations) and chemical (ozone) treatments on cysts in a simple matrix, and to assess the exposure of humans to infective cysts in naturally-contaminated samples (Table 1).

#### 2.2.3 Toxoplasma gondii

A review of the relationship between seroprevalence in the main livestock species and presence of *T. gondii* in meat analyzed the data from studies providing a direct comparison of two or more direct detection methods into a performance matrix [171]. It was clear from this that the cat bioassay performs best, followed by the mouse bioassay. However, the bioassay in cats is performed in very few laboratories because of the difficulty in implementing an assay with considerable associated ethical issues; therefore, Swiss Webster and SCID mice are the main animal models to assess infectivity of *T. gondii* oocysts [54,56,119], SCID mice being the most sensitive. Mice are tested for *T. gondii* seroconversion four weeks post-infection and brain preparations are then examined for tissue cysts by microscopy [219]. Such models have been used to evaluate the presence of infective oocysts in water and shellfish samples (Table 1), and to estimate infectivity of oocysts in soil [136]. Bioassays were also successfully used to evaluate the efficacy of different treatments (e.g., oxidants, UV, high hydrostatic pressure) and the impact of storage time and temperatures on the infectivity of oocysts in simple matrices or adhering to the surface of raspberries and blueberries (Table 1).

To summarize, animal models have been reliably used to evaluate the exposure of humans to infective parasites and can be applied to foods. However, these methods suffer from limitations, the major one being ethical concerns, but also definition of a suitable animal model, lack of reliability when low numbers are used, and expense (Table 3). These disadvantages challenge the scientific community to develop and investigate alternative methods to accurately assess infectivity.

**Table 3:** Advantages and limitations of the techniques used to assess infectivity and viability.

Methods	Parasites	Advantages	Limitations
Bioassays [I]	Cp & Ch / G / T	- Requires a single (oo)cyst - Application on complex food matrices - Reliable exposure assessment of humans to infective protozoa. − Reliable assessment of inactivation efficacy	- Ethical concerns - Expensive method and labor intensive - Lack of reliability with low numbers of (oo)cysts - Discrimination of large but not fine differences between treatments - Long time-to-results (one week minimum)

Cell Culture [I]	Cp & Ch / T	- Correlation with bioassays - Requires a single (oo)cyst - Reliable exposure assessment of humans to infective protozoa - Reliable assessment of inactivation efficacy	- Lack of standardized assays - Medium (48/72h) to long (10 days) time to results - Not applicable to *Giardia* - No application on complex food matrices

Methods based on morphology and physical properties [V]	C / G	- Correlation between electrorotation and PI exclusion method - Rapid - Require a single (oo)cyst	- Require purified (oo)cysts - No data on viability relative to infectivity measure - No application in exposure assessment in food

Excystation [V]	C / G / T	- Correlation with PI exclusion method - Rapid - Non-expensive	- Requires large numbers of purified (oo)cysts - Variable excystation rate - Over/underestimation of the exposure of humans to infective protozoa* - Over/underestimation of inactivation efficacy* - No application on complex food matrices

Vital dye exclusion [V]	C / G / T	- Correlation with excystation for PI dye (C) - Rapid and relatively simple - Non-expensive - Requires a single (oo)cysts - Application on complex food matrices	- Requires purified (oo)cysts - Few applications for *T. gondii* - Overestimation of the exposure of humans to infective protozoa* - Underestimation of inactivation efficacy*

RT-PCR [V]	C / G / T	- Rapid - Identification of (oo)cysts of human health significance - Easy to standardize - Requires low numbers of (oo)cysts - Application on complex food matrices	- Limit of detection variable depending on matrices - Overestimation of the exposure of humans to infective protozoa* - Underestimation of inactivation efficacy*

FISH [V]	C	- Rapid - Non-expensive - Requires a single (oo)cysts	- Requires purified (oo)cysts - Overestimation of the exposure of humans to infective protozoa* - Underestimation of inactivation efficacy* - No application on complex food matrices

NASBA [V]	C	- Correlation with PI exclusion method - Rapid - Non-expensive - Requires low numbers of (oo)cysts	- Overestimation of the viable population - Non-quantitative - No data on viability relative to infectivity measure - No application in exposure assessment in food

PMA-PCR [V]	C	- Rapid - Requires low numbers of (oo)cysts - Can be coupled to genotyping - Easy to standardize	- Overestimation of the viable population - No data on viability relative to infectivity measure - No application in exposure assessment in food

[I]: infectivity. [V]: viability. C: *Cryptosporidium *spp.; Cp: *C. parvum*; Ch: *C. hominis*; G: *Giardia duodenalis*; T: *Toxoplasma gondii*.

* Based on the comparison of viability relative to infectivity.

### 2.3 Cell culture infection

#### 2.3.1 Cryptosporidium spp.

Investigators have turned to cell cultures as a model for *Cryptosporidium* infection to avoid the problems associated with animal models. Multiple cell lines support *Cryptosporidium* replication, and complete life cycle development has been established in several of them [35,214]. Human cell lines are of particular interest, since they can support the development of *C. parvum* and *C. hominis* parasites. These cell lines are highly relevant for human infections as oocyst infectivity and dose-response relationships can easily be studied, and some aspects of *Cryptosporidium* pathogenicity, such as cell damage and barrier permeability, can also be examined. Human enterocytes HCT-8 are currently the model of choice for evaluating oocyst infectivity because they are relatively easy to maintain and support infection with low oocyst numbers [214]. Moreover, the results obtained with HCT-8 cell lines have been shown to have a better correlation with the neonate BALB/c and CD-1 mouse models than those obtained with Caco-2 and MDCK cell lines [187,198]. A non-carcinoma, human small intestinal epithelial cell type, named FHs 74 Int has been described as exhibiting higher levels of infection susceptibility compared with other cell types [216]. A recent study described a further cell line (COLO-680N) as a cell-culture platform that is easy-to-handle and enables the long-term sustainable production of infective oocysts at a laboratory scale, and is presumed will provide a valuable tool for viability assessment as well as other investigations [159].

Cell cultures can be inoculated with whole purified oocysts, mixtures of intact and excysted oocysts, and free or purified sporozoites. Numerous studies have tried to improve the success of cell culture assays by pre-activation of oocysts with bile salts [124], improving culture medium composition [122,213], and favoring sporozoite-host cell contact by centrifugation before co-culture [125]. Compared with PCR and RT-qPCR [51,133], fluorescent labeling and enumeration of developing parasitic foci in cell monolayers is one of the simplest and the most reliable ways to assess oocyst infectivity [117,190,197]. It has been shown that an electrical impedance-based device is able to provide insights on *Cryptosporidium* development in HCT-8 cell cultures. By quantifying the magnitude of the impedimetric response, this device can be used as an infectivity sensor as early as 12 h post-infection [52]. Cell culture assays have been reported to achieve a sensitivity of one single infectious oocyst and are unlikely to be subject to false-positive results [117]. However, no data are available for species other than *C. parvum* and *C. hominis*.

Cell-culture assays have been used to assess the exposure of humans to infective *Cryptosporidium* spp. oocysts in various types of environmental water samples (Table 1), although in the most recent large waterborne outbreaks of cryptosporidiosis, they do not appear to have been applied [22,50]. This approach also seems relevant to assess the efficacy of control measures that can be used in food industries (i.e. oxidants, organic acids, heating or storage at 2-8 °C) in simple matrices and in fruit juices, but it has not been extensively applied in foods (Table 1).

#### 2.3.2 Giardia duodenalis

Two studies have described *in vitro* cell culture of *G. duodenalis* combined with PCR or RT-PCR assay [8,84]. However, as *Giardia* is not an intracellular parasite and can be successfully cultivated in the absence of other cells (i.e., axenic culture), the value of such an assay to assess the infective potential is unclear.

#### 2.3.3 Toxoplasma gondii

*Toxoplasma gondii* parasites can multiply in virtually any nucleated cell types; however, only murine L20B fibroblasts and human foreskin fibroblasts (HFF) cells have been used to determine the impact of UV, oxidants and other chemicals on oocysts in simple matrices, combined with microscopy to detect tachyzoite foci (Table 1). To date, there seems to have been no application of this method to food matrices.

In conclusion, cell culture appears to be a good alternative method to bioassays for those parasites that are intracellular, showing a good correlation with infectivity for both *Cryptosporidium* and *T. gondii* oocysts. Thus, this technique has the potential to provide reliable estimates of exposure to infective protozoa from field to fork. Nevertheless, application of this method on complex food matrices has still not been described (Table 3). Due to its shorter time-to-results than bioassays and its reported ability to detect as few as one infective oocyst (*C. parvum*), this method should be of interest in prospective occurrence investigations. However, for retrospective investigations, when hazards are detected in foods or in case of foodborne outbreaks, more rapid results are essential so that contaminated foods can be removed from the market.

## 3 Evaluation of viability

Evaluation of viability means determining whether (oo)cysts are alive. However, living, intact, metabolically functional (oo)cysts are not necessarily infective, because their trophozoites/sporozoites can fail to cause infection of their hosts. Hence, to obtain a reliable assessment of the exposure of humans to infective parasites, methods to measure viability are of greatest use if they correlate with the results from infectivity assays. If such a correlation is not established, then detection of viable (oo)cysts does not mean that infective parasites are present, and thus any risk may be overestimated. It thus follows, in using such an assay to determine the efficacy of a treatment process, the effectiveness of the process may be underestimated or the process may be wrongly considered non-effective. In contrast, if false negatives are found, or the method has a poor recovery performance, the population of infective (oo)cysts may be underestimated. In these latter cases, the efficacy of a particular process could be overestimated, and the subsequent risk posed to the population underestimated. In terms of food safety, it is generally considered preferable to use an assay that may overestimate the risk (and underestimate process efficacy), than the other way around, as this conservative approach (“worst-case scenario” approach) ensures that the public health risk is minimized.

### 3.1 Changes in morphology and physical properties

Any visible modification in the morphology, wall integrity, and internal content of the cysts and oocysts may indicate a loss of viability at the single parasite level. Cysts and oocysts with shape deformation, openings in their walls, and/or filled with a granular content with no evidence of intact sporozoites (*C. parvum* and *T. gondii*) or internal bodies (*G. duodenalis*), likely represent non-viable parasites that can be observed microscopically [215]. Non-invasive methods have been described to determine the viability of the oocysts and cysts as a function of their size, morphology and surface electric charges. For instance, electrorotation is a qualitative technique that can be used to detect changes in the morphology and physicochemical properties of microorganisms subjected to an electric rotating field. This approach has been successfully used to investigate the viability of *C. parvum* [86,87] and *G. intestinalis* [47], and the sporulation state of *Cyclospora cayetanensis* which is closely related to *T. gondii* [47]. More recently, microfluidic devices have been designed to measure the size and deformability of *C. parvum* and *C. muris* oocysts by force spectroscopy [153]. This method has been reported to reliably discriminate *C. parvum* from *C. muris* oocysts, and viable from heat-killed *C. parvum* oocysts based on the deformability properties of the oocysts. Such methods could be useful for evaluating the viability of *C. parvum* oocysts in environmental samples with low backgrounds, such as drinking water. However, the use of these methods has not yet been widely adopted, if at all, and their use in the framework of exposure assessment in foods has not been investigated. As the structure and physico-chemical properties of (oo)cysts may be modified in the environment, including in food matrices, and following stressing treatments [160], without affecting viability, it seems unlikely that electrorotation and other biophysical methods will develop beyond being research tools.

### 3.2 Excystation assays

It is generally assumed that parasites able to excyst *in vitro* are viable and likely to be infectious. In order to induce excystation and release of sporozoites (*Cryptosporidium* and *T. gondii)* and trophozoites (*Giardia*), several treatments have been described that are based upon mimicking the conditions that the parasite would encounter *in vivo* in a susceptible host. Thus, *C. parvum* oocysts can be incubated at 37 °C in solutions containing trypsin with or without sodium taurocholate, or surfactants (sodium dodecyl sulfate) [36,66,178], but excystation rates can vary depending on the dose, time and pre-treatment temperature [124]. Pre-treating *C. parvum* oocysts with acidic and sodium hypochlorite solutions can enhance excystation of the sporozoites [36,184]. An excystation assay has also been described for *C. muris* [157]. Due to the additional presence of sporocysts in *T. gondii* oocysts, sporozoite excystation first requires opening of the oocyst wall, which is usually achieved *in vitro* by physical means (sonication or bead beating), then disruption of the sporocyst wall following incubation in digestive solution containing bile salts [79]. Standard protocols for excystation of *Giardia* trophozoites from cysts have been available for many decades [25,26,100,181] and tend to use two sequential steps, a low-pH induction step (using, for example, acidified saline solutions at pH 2-2.7, often supplemented with L-cysteine hydrochloride and glutathione), followed by an excystation step in which the cysts are exposed to a medium such as trypsin in Tyrodes solution at pH 8. Bile supplementation may also be used. However, successful *in vitro* excystation is assemblage-dependent, and researchers often struggle to achieve excystation when working with non-laboratory adapted field strains [211].

Excystation is measured as the percentage of excysted (oo)cysts determined by phase contrast microscopy, or qPCR assays [173]. In order to differentiate between excystation and “bursting” of the oocysts due to stress, it is important, for *Cryptosporidium* at least, that active sporozoite ratios are also determined. Sporozoites that have failed to excyst can be visualized inside the oocysts with a DNA binding dye, such as 4’, 6-diamidino-2-phenylindole (DAPI) [36]. The excystation method requires a large number of purified (oo)cysts, and an insufficient number may occur when investigating naturally contaminated samples. Consistent with this, only one study reported the use of excystation assays to assess exposure to infective *C. parvum* in drinking water (Table 2). In most studies evaluating the impact of oxidants or UV on *Cryptosporidium* and *Giardia* (oo)cysts, excystation does not correlate with infectivity assays (Table 2). Indeed, the ability of the parasites to excyst does not necessarily mean that the parasites will complete their development in the host. Thus, excystation methods overestimate the exposure of humans to infective parasites, and treatments that do not kill the parasites but cause them to become no longer infectious to cells, may have their inactivation credit underestimated. However, in contrast*, in vitro* excystation may also overestimate inactivation efficacy should substantial numbers of viable (potentially infectious) (oo)cysts fail to excyst when subject to a particular protocol, but nevertheless still remain infective for animals [108]. The same overestimation could also occur should excysted sporozoites of *C. parvum* rapidly lose their viability *in vitro* [152].

**Table 2:** Methods to determine (oo)cyst viability and their application in exposure assessment.

Methods	Parasites	Applications			

Detection in naturally contaminated samples	Agreement with infectivity assays	Determination of the efficacy of control measures^a^	Agreement with infectivity assays
Excystation Ability of trophozoites / sporozoites to escape from the cysts / oocysts after stimulation	Cp	Drinking water [80]	NA	**Simple matrix^b^:**	
				H_2_O_2_-based disinfectants [176]	–
				Chlorine [29], Chlorine dioxide, Monochloramine [129]	–
				Ozone [29,34,73,129]	–
				Ammonia [116]	–
				Liming, alum and ferric sulfate floccing [183]	NA
				UV [77,89,123,154,158]	−
				Freezing [183]	NA
				Storage at 4 °C and 10 °C (8 w) [142]	–
				Dessication [183]	NA
	G	NA	NA	**Simple matrix:**	
				Vinegar (acetic acid 4%) à 4 °C et 21 °C [45]	NA
				UV [84]	–
				Storage at 4 °C (up to 56 d) [25]	NA
Vital dye-exclusion (propidium iodide) Ability of cells to exclude the dye (membrane intact cells) & staining of dead cells	Cp	Wastewater [6]	NA	**Simple matrix:**	
		Surface water [190]	− ^§^	H_2_O_2_-based disinfectants [176]	–
		Marine water [191]	NA	Chlorine [29]	–
		Drinking water [6]	NA	Ozone [29,34]	–
		Cockles, mussels, clams [90]	NA	Ammonia [116] Liming, alum and ferric sulfate floccing [183]	– NA
		Oysters [90,191]	NA	UV [77,89,154,158]	+/−
				Freeze-thaw [121]	NA
				Freezing [183]	NA
				Storage 4 °C and 20 °C (12 w) [166]	NA
				Dessication [183]	NA
	G	Drinking and wastewater [6]	NA	**Simple matrix:**	
				UV [37,84,162]	–
				Gamma radiation [203]	–
				Storage 4 °C (up to 56 d) [24]	NA
RT-PCR Ability of cells to produce mRNA	Cp	NA	NA	**Simple matrix:**	
				H_2_O_2_ [*hsp70*] [144]	NA
				Chlorine, Chlorine dioxide, MOS [*hsp70, β-tubulin*] [15,97]	–
				Ozone [*hsp70*] [97]	NA
				Ammonia [*hsp70*] [144]	NA
				Heat 60 °C to 95 °C [*hsp70*] [97,209]	–
				Freeze [*hsp70*] [97]	NA
				Storage 15 °C (9 m) [*cpag*] [114,115]	+/−
				Storage 4 °C (48 m) [*hsp70*] [144]	NA
				Storage RT and 4 °C (20 to 39 w) [*β-tubulin*] [226]	+
				**Food matrix:**	
				Storage 4 °C (8 d) − Basil [*hsp70*] [104]	NA
	G	Wastewater [17,24]	NA	**Simple matrix:**	
				Heat 60 °C to 95 °C [*β-giardin*] [209]	–
				**Food matrix:**	
				Storage 4 °C (8 d) − Basil [*β-giardin*] [104]	NA
	T	NA	NA	**Simple matrix:**	
				Chlorine-based disinfectant [*sporoSAG, ACT1*] [218]	+
				Acidified ethanol and iodophore-based disinfectant	+
				[sporoSAG, ACT1] [218]	
				Formalin [*sporoSAG, ACT1*] [218]	–
				UV [*sporoSAG, ACT1*] [223]	–
				Heat 60 °C to 80 °C [*sporoSAG, ACT1*] [209,218]	–
				**Food matrix:**	
				Storage 4 °C (8 d) − Basil [*sporoSAG*] [104]	NA
FISH Ability of cells to produce rRNA	Cp	Water [138] Oysters [93]	NA NA	**Simple matrix:** Gamma irradiation [185]	NA
				Storage 15 °C (9 m) [93]	–
	G	Water [138]	NA	NA	NA
PMA-PCR Ability of cells to exclude PMA (membrane intact cells) & no mplification in dead cells	Cp	Water [149]	NA	**Simple matrix:**	
				H_2_O_2_ [144]	NA
				Ammonia [144]	NA
				Heat 70 °C [33]	NA
				Storage at RT (14 m) [33]	NA
				Storage 4 °C (up to 48 m) [*hsp70*] [144]	NA
	G	Water [149]	NA	NA	NA

a Relevant for water and food industries;

b Simple matrix: water or buffer.

NA: No available data. Cp: *C. parvum*; G: *Giardia* *duodenalis*; T:*Toxoplasma* *gondii*.

H_2_O_2_: hydrogen peroxide; UV: ultraviolet; RT: room temperature; m: months; w: weeks; d: days.

(−): no agreement with infectivity assays means that: i) viability and infectivity assays are not concordant (i.e. viability (+) and infectivity (−), and conversely (rarely)); ii) and/or lower inactivation levels or no inactivation are measured by viability assays compared to infectivity assays.

(+): viability and infectivity assays are concordant.

(+/−): agreement with infectivity varies according to studies

§: 12/15 samples showed no correlation with cell culture assays.

To conclude, despite there being a good correlation with the viability measure based on vital dyes exclusion (PI), excystation assays have many limitations and cannot be considered as an appropriate method to assess the risk of infection associated with exposure to parasites detected in food (Table 3).

### 3.3 Viability assays using dyes for live and dead cells

A commonly used approach to assess viability is to use inclusion and exclusion of specific dyes, often known as vital dye assays, as colored markers of viability. The dye inclusion or exclusion can be evaluated by microscopy (fluorescence microscopy, if the dye used is fluorescent). As each cell (or parasite) can be coded individually according to its dye uptake/activation or exclusion, the assay can be applied to individual parasites or low numbers. If large numbers of parasites are used, then flow cytometry or cell sorting can be used [164]. Relatively few experiments have investigated the use of such dyes for investigating the viability of *Toxoplasma* oocysts, and the double walls of oocysts and sporocysts indicate that uptake of dyes into these oocysts may be more complex. In addition, the characteristic autofluorescence occurring with *T. gondii* oocysts may act as a confounder [58].

#### 3.3.1 Dye exclusion methods

These methods are based on dyes that can penetrate selectively into cells that have lost their membrane integrity and are excluded by live cells, thus these dyes stain parasites that are dead. Dyes that have been used successfully for this assessment include non-fluorescent dyes such as trypan blue [202], and fluorescent dyes such as eosin, ethidium bromide (EB), ethidium monoazide (EMA), propidium monoazide (PMA), and propidium iodide (PI). EB has been extensively used as a DNA intercalating agent, emitting red fluorescence in the dead cells [229], as well as PI. EMA and PMA, both similar to EB and PI respectively, display an azide group, allowing cross-linkage to DNA upon light exposure, but the main limit of EMA is that it can penetrate into intact cells [168].

Trypan blue stain has been used to assess the effect of household disinfectants on protozoan parasites contaminating fresh produce, including *Giardia* cysts and *Cryptosporidium* oocysts, with preliminary results supported by infection studies [62]. PI has been successfully applied to assess the viability of *C. parvum* oocysts in such complex food matrices as shellfish [78]. This dye probably remains the most commonly used and thus, only studies referring to PI are reviewed in Table 2. Whereas PI assays have been shown to correlate with *in vitro* excystation of *C. parvum* oocysts [36], and have been widely used in studies in which natural die off in the environment is measured, for investigation of the use of oxidants and UV, PI staining of *C. parvum* and *Giardia* (oo)cysts in simple matrices tends not to correlate with infectivity (Table 2). This is probably due to the method by which these decontamination treatments interact with the parasite DNA.

One major limitation of dye-exclusion approaches is that a cell can have an intact membrane but will nevertheless be non-viable. Thus, parasites that are non-viable but have an intact membrane will not stain with those dyes. Hence, dye-exclusion technique leads to the overestimation of the exposure of humans to infective parasites and to the underestimation of the efficacy of control measures.

#### 3.3.2 Dye inclusion/exclusion methods

In order to overcome some of the deficiencies of the dye exclusion technique, inclusion-exclusion methods have been developed in which viable cells, as well as excluding specific dyes, also include specific dyes, and the dyes are used simultaneously in an inclusion-exclusion assay. One approach is to incubate cells with a non-fluorescent dye. These diffuse into living cells and then, following cleavage by intracellular enzymes present only in live cells, a fluorescent molecule is produced which is then detected. One such dye is fluorescein diacetate (FDA), which is nontoxic for many cell types [189], and is a substrate for a cell-permanent esterase leading to the production of fluorescein, which accumulates in live cells only if their membrane is intact, and exhibits green fluorescence when excited by blue light, indicating a viable cell. This was suggested to be used in conjunction with PI as an exclusion-inclusion assay for *Giardia muris* cysts, as a model for *G. duodenalis* [192]. Unfortunately, however, further investigation revealed that *G. duodenalis* cysts that stained with PI could also often stain with FDA [199], presumably because of the enzyme remaining active despite the cyst itself no longer being viable. A similar approach has also been followed with the redox dye 5-cyano-2,3-ditolyl tetrazolium chloride (CTC), that should only show staining when the respiratory electron transport system (ETS) shows activity (i.e., the parasite is viable). Although initial studies provided promising results [113], the same type of problem of activity even in dead cells is likely to limit the usefulness of this approach. An alternative approach to these non-fluorescent dyes is the use of dyes that are nuclear intercalates such as SYTO-9, SYTO-17, SYTO-59-64 and hexidium, and DAPI. SYTO-dyes seem to provide viability assay results that correlate with the infectivity of *C. parvum* oocysts to CD-1 mice but not to excystation assays [19,20,165] assessing the efficacy of oxidants, UV and thermal treatments. That said, recent experiments have shown that of seven SYTO dyes investigated, all were more likely to stain dead *Cryptosporidium* oocysts than live oocysts [208], indicating some degree of confusion around use of vital dye stains.

In conclusion, there is considerable confusion and potential for subjectivity regarding dyes used for inclusion. Vital dye exclusion overestimates the exposure of humans to infective protozoa, suggesting that this technique may not be appropriate for exposure assessment. However, given its applicability to foods, its suitability for low numbers of parasites, and its cost-effectiveness, among others advantages (Table 3), it may provide initial results, particularly when investigating whether individual parasites identified on fresh produce are potentially infectious or whether a treatment is efficient to kill (oo)cysts.

### 3.4 Molecular methods

#### 3.4.1 RNA-based methods

##### 3.4.1.1 Reverse Transcriptase-PCR (RT-PCR)

RT-PCR techniques are emerging as alternatives to the use of vital dyes for evaluating viability. These methods are based on the production of mRNA in cells that are metabolically functional and active, and hence considered as viable.

###### 3.4.1.1.1 Cryptosporidium parvum

For *Cryptosporidium* oocysts, RT-PCR assays targeting the *hsp70* gene have been described as being highly sensitive. Indeed, the level of *hsp70* mRNA can be enhanced by heating. This allows increasing the sensitivity especially when working on initial low levels of mRNA or in complex matrices [81]. Other targets have also been described, such as genes encoding *β-tubulin* [226], *amylogluconidase* [114], *COWP*, *CP2* or 18S rDNA [135]. However, 18S rRNA remains stable for a prolonged period (up to at least 48 h) in heat-killed oocysts, and hence is not a good marker of viability [76,135]. RT-PCR was used to assess the efficacy of temperature and chemicals on *Cryptosporidium* oocysts in simple and complex matrices (Table 2). However, correlation with infectivity seems to depend on the applied treatments and the targeted mRNA: *β-tubulin* mRNA production was reported to correlate with oocyst infectivity after long-term storage at cold and room temperatures but not following oxidant treatments, while *hsp70 *mRNA production correlated with cell culture assays in oocysts submitted to oxidants, but not to heat treatment (Table 2).

###### 3.4.1.1.2 Giardia duodenalis

The *hsp70* gene has also been described for the detection of viable *Giardia* cysts in simplex assays [1,134], but also in duplex RT-PCR with *C. parvum* [163]. Other targets such as *ef1a,* *adhe* and *β*-*giardin* mRNAs have been used, the latter being the most suitable to study the viability of *Giardia* cysts in environmental samples (Table 2). However, *β-giardin* mRNA RT-PCR assays have been shown to detect viable but non-infectious cysts following heat treatments, indicating that this method underestimates the efficacy of these treatments (Tables 2 and 3).

###### 3.4.1.1.3 Toxoplasma gondii

To estimate the viability of *T. gondii* oocysts, RT-PCR assays targeting two genes have been described: *SporoSAG*, which is highly expressed in the sporulated oocyst, and *act1* expressed in both sporulated and unsporulated oocysts [218]. As previously described for *Cryptosporidium*, correlation of RT-PCR with infectivity assays appears variable according to the applied treatments, with good agreement following chlorine- and iodine-based disinfectants, and acidified-ethanol, but not after heating or exposure to formalin or UV (Table 2).

To summarize, the RT-qPCR technique is rapid, sensitive, and can directly assess the likelihood for a human to be infected by detected protozoa. However, although mRNA is the least stable nucleic acid, compared with rDNA, tRNA and rRNA, it can nevertheless persist for a long time after the death of the parasites, even in non-favorable conditions. This has recently been described for mRNA from dead *Plasmodium* ookinetes that remained detectable more than 24 h inside the mosquito midgut [96]. Hence, RT-qPCR assays can overestimate the number of viable and potentially infectious (oo)cysts, and consequently will overestimate the exposure of humans to infective parasites and underestimate the efficacy of control measures (Table 3). However, this method can be useful as a first step of screening of different treatments applied to complex matrices to benchmark inactivation potential.

##### 3.4.1.2 Fluorescence *in situ* hybridization (FISH)

The fluorescence *in situ* hybridization (FISH) method consists in targeting a nucleic acid sequence using a specific synthetic fluorescent oligonucleotide probe. rRNA is considered to be the ideal viability target for several reasons: (i) high sensitivity; (ii) short half-life (although as described in 3.4.1.1, in parasites the half-life is longer); (iii) present in high copy number in viable cells [9]. Hence, a FISH ’positive’ signal depends on the amount of target rRNAs present in a cell, the longevity of that target within the cell and accessibility for probing, the fluorescence intensity of bound probe and the presence of RNases. Probes targeting different regions of the 18S rRNA specific to *C. parvum*, *C. hominis* and *G. duodenalis* have been described for the detection of viable (oo)cyst [5,48,92,217]. The FISH technique is described as a rapid and cheap method, with a fast time-to-result (3 hours), enabling the specific simultaneous visualization of one or more viable (oo)cyst species [92,138]. Nevertheless, it remains scarcely used in surveys (Table 2). Although in some studies FISH assays have shown correlation with *in vitro* excystation of *C. parvum* oocysts [217], this is not always the case and no agreement with infectivity assays has been observed [115]. Furthermore, rRNA is able to persist for 6 days after *C. parvum* oocyst heat treatment, presumably because it is protected within the oocyst [200], and consistent with this, gamma-irradiated oocysts of *Cryptosporidium* have been shown to give a positive FISH signal while being dead (Table 2). Thus, this method cannot be considered as a reliable measure to assess the exposure of humans to infective (oo)cysts and the inactivation efficacy of treatments (Table 3).

##### 3.4.1.3 Nucleic Acid Sequence-Based Amplification (NASBA)

Nucleic acid sequence-based amplification (NASBA) is an isothermal and sensitive technique for amplification of RNA targets that has been described for many microorganisms and has recently been reviewed [106]. This method can detect viable cells through selective amplification of mRNA, even in a background of genomic DNA. Compared with RT-qPCR techniques, amplification is performed at a single temperature (41 °C), and consequently does not need thermal cycling equipment. The detection of products of NASBA reaction is quite labor-intensive including ethidium bromide-stained agarose gel electrophoresis, which requires a confirmatory step by probe hybridization, enzyme-linked gel assays, electrochemiluminescence (ECL) detection and fluorescence correlation spectroscopy. *MIC1* and *hsp70* mRNAs have been described as target molecules for *Cryptosporidium* oocysts [13,107]. This method has been shown to correlate with the PI exclusion method [44] and to be suitable to detect viable *C. parvum* oocysts in spiked environmental waters, in the presence of organic, inorganic and biological contaminants, and with detection limit below ten viable oocysts per analyzed sample [13]. However, oocysts that had been killed by exposure to sodium hypochlorite, freeze-thawing or boiling were also detected, indicating again the stability of the RNA and the lack of suitability of this method for detecting only viable oocysts; indeed, freeze-thawing seemed to give an even stronger NASBA signal [107]. To date, no study describes the use of this method to evaluate the occurrence of parasites in naturally contaminated samples and to assess the efficacy of industrial processes on the protozoan parasites, and no such assays have been proposed for *T. gondii* or *G. duodenalis*.

In conclusion, because of the stability of mRNA for extended periods in oocysts that have been experimentally inactivated (e.g., after heating, freeze-thawing or chemical treatments), it is clear that this technique detects both live and dead oocysts. Moreover, although quantitative NASBA has been described, the cell concentration seems not to be reliable, and thus this method is unsuitable for inactivation studies. Therefore, this technique appears not to be appropriate for exposure assessment in food (Table 3).

#### 3.4.2 DNA-based methods: EMA/PMA PCR

Combination of viability dyes like EMA and PMA with PCR has been developed to study the viability of different foodborne pathogens [231]. As described in the “dyes” section (3.3.1), the use of EMA and PMA in viability assessment is based on the impermeability of live cells to both dyes that penetrate damaged and permeable cell (considered as non-viable) only. Once inside the nucleus, EMA and PMA can be photoactivated, which leads to their covalent binding to DNA and prevents PCR amplification in non-viable cells [30,75]. Thus, a PCR signal should be obtained only for viable cells (i.e. non-damaged and non-permeable cells). However, in contrast with PMA, EMA has been described as able to penetrate into viable cells due to the variability of cell membrane structure and of active membrane transport efficacy [75,168]. EMA is also considered more toxic than PMA and this toxicity is time and temperature dependent [75]. Hence, PMA should lead to better discrimination of viable cells than EMA.

PMA-PCR and PMA-qPCR targeting the *hsp70*, *COWP* and 18S rRNA encoding genes have been described for *Cryptosporidium* oocysts [3,7,33,144]. For *Giardia*, PMA-qPCR based on the detection of the *b-giardin* gene has been shown to be able to quantify viable cysts in artificially contaminated wastewater samples accurately. This target has appeared as more effective than the triosephosphate isomerase (*tpi*) and glutamate dehydrogenase (*GDH*) genes [7]. In this study, the authors have also shown that the longer the amplicon, the more effective the exclusion of dead cysts. Comparison with infectivity assays has not been performed, and PMA-qPCR appears not to correlate with RT-qPCR data (unpublished data). Only a few studies have described the use of PMA-PCR to identify viable parasites in naturally contaminated water samples, and to assess the efficacy of chemicals and temperature on *Cryptosporidium* oocysts and *Giardia* cysts (Table 2). Up to now, no study has described PMA-qPCR applied to *T. gondii*. However, the ability to specifically detect viable protozoa seems to depend on the applied treatment and is impaired in complex samples (unpublished data). Thus, this technique may overestimate the exposure to infective protozoa.

## 4 Conclusion

To date, only techniques allowing infectivity evaluation are able to provide a reliable assessment of the exposure of humans to protozoa in food (Table 3). They can even be sufficiently sensitive to detect low levels of contamination and directly address the likelihood that individuals will be infected by consumption of contaminated food. On the one hand, bioassays can be directly applied to food samples but they suffer from major ethical concerns; additionally they are restrictive, expensive, and implementation is complex, with prolonged time-to-results. Hence, bioassays are not suitable for routine analysis for control measure verifications or any surveys intended to assess the global exposure of consumers. On the other hand, cell cultures can produce results more rapidly (but still 2 to 10 days), and this method could be more available and appropriate for routine laboratories (but still requiring specific skills, including long-term cell culture). However, it requires purified (oo)cysts, which can be challenging to obtain when working on food matrices. Both methods produce reliable inactivation data, but some authors have suggested that bioassays and cell culture may be able to highlight only substantial inactivation.

Most techniques assessing (oo)cyst viability appear to overestimate the occurrence of infective parasites and subsequent exposure of humans along the food chain. When used for investigating the efficacy of control measures, they tend to underestimate their inactivation efficacy (Table 3). RNA-amplification based assays (RT-PCR, NASBA) are interesting alternative techniques because they can be standardized and may be suitable for routine analyses of food samples (implementation and results interpretation). Moreover, being a quantitative approach, RT-qPCR allows the definition of viability reduction levels. However, these positive aspects are undermined by the apparent and clear stability of RNA within parasites, such that dead parasites are likely to be considered viable, overestimating exposure of humans to infective parasites and underestimating the efficacy of control measures. Although PMA-PCR seems to be an exciting and relevant technique, unfortunately it is only limited to simple matrices and to the evaluation of control measures that would permeabilize the (oo)cysts and allow the dye to penetrate. Excystation assays provide an active measure of activity and the effect of an intervention, but require clean suspensions and high numbers of parasites to be of any use. Their application beyond experimental studies of *Cryptosporidium* oocysts seems limited, and they are not easy to standardize in routine laboratory analyses. Although vital dye assays have considerable limitations, particularly with respect to overestimation of viability due to membrane impermeability not necessarily correlating with viability, and the subjectivity of any microscopy-based technique, their simplicity, cost-effectiveness, and potential for application to low parasite numbers may make them useful for initial screening purposes − both with regard to assessing infection potential of contaminant parasites and investigating treatment effects. However, results should always be backed up with another technique, and their disadvantages should not be overlooked.

To conclude, none of the techniques currently available appear to be entirely suitable for reliable assessment of the exposure of humans to infective protozoa in food or for routine verifications of control measures. In order to be able to produce data to refine exposure and subsequently better characterize the risk, we suggest that two actions should be carried out in parallel:

- i) Determine initial levels of contamination using DNA-based assays: these are known to be sensitive enough for detecting low quantities of parasites, are easy to standardize, and are accessible for routine analyses. Although false positives are not expected, their potential should not be overlooked due to the occurrence of “loose” target DNA, without the actual organism being present. These methods overestimate the exposure to infective parasites by detecting all populations of (oo)cysts (i.e. live and infectious, live and non-infectious, dead), and therefore will give the maximum occurrence and level of contamination for a given matrix. For *Cryptosporidium* and *Giardia,* determination of the species and the genetic assemblages respectively, is part of the exposure assessment. These important taxonomic details can be determined by DNA-based assays and directly address the infectious potential for humans.
- ii) Characterize the efficacy of treatments in place to control parasites using a combination of techniques. Simple vital dye inclusion/exclusion methods may be the preliminary approach, providing initial data on the inactivation potential of different treatments. To determine inactivation levels, which are the reference measure for food operators (log_10_ viability reduction), RT-qPCR-based assays could be applied. They will overestimate the level of infective protozoa (quantification of live and infectious, live and non-infectious, and possibly dead (oo)cysts), leading to an underestimation of the efficacy of the treatments. But this action will allow the determination of the “worst case scenario” for a specific combination food/process, providing at the same time, confidence that when a target inactivation is reached, higher inactivation occurs in reality. Cell culture methods are improving in applicability, and, as imaging techniques become more accessible and cheap, will be of value in assessing invasion of cell cultures. Appropriate infectivity assays (bioassays), should be minimized for ethical and operational reasons, but can be used to confirm results obtained by *in vitro* assays.

All viability assays are based on evaluation of distinct physiological parameters that could vary depending on the applied treatments, the protozoa in question, and their original physiological state. Hence, future research should focus on the characterization of the effect of inactivation methods on the structure and the metabolism of (oo)cysts to be able to select and develop suitable techniques for infectivity measurement.

## Conflict of interest

The authors declare no conflict of interest.
